# COVID-19 viral load not associated with disease severity: findings from a retrospective cohort study

**DOI:** 10.1186/s12879-021-06376-1

**Published:** 2021-07-16

**Authors:** Abdulkarim Abdulrahman, Saad I. Mallah, Manaf Alqahtani

**Affiliations:** 1 National Taskforce for Combating the Coronavirus (COVID-19), Manama, Bahrain; 2 Mohammed Bin Khalifa Cardiac Centre, Awali, Bahrain; 3 Royal College of Surgeons in Ireland, Al Sayh, Bahrain; 4 Bahrain Defence Force Hospital, Riffa, Bahrain

## Abstract

**Background:**

Being able to use COVID-19 RT-PCR Ct values as simple clinical markers of disease outcome or prognosis would allow for the easy and proactive identification and triaging of high-risk cases. This study’s objective was thus to explore whether a correlation exists between COVID-19 viral loads, as indicated by RT-PCR Ct values, and disease severity, as indicated by respiratory indices.

**Results:**

A multi-centre cross-sectional retrospective study was conducted, using data obtained from Bahrain’s National COVID-19 Task force’s centralised database. The study period ranged from May 2, 2020 to July 31, 2020. A multivariable logistic regression was used to assess for a correlation using data from a total of 1057 admitted COVID-19 cases. The covariates adjusted for included sex, age, presentation, and comorbidities. In our cohort, Ct value showed no statistical significance for an association with requirement for oxygenation on admission (Odds ratio 1.046; 95%CI 0.999 to 1.096, *p* = 0.054).

**Conclusion:**

Viral load, as indicated by Ct values, did not seem to be associated with requirement for oxygenation on admission in our cohort. We postulate however that time since onset of symptom may have acted as an unaccounted-for confounder. As such, RT-PCR Ct values may not be a useful prognostic clinical tool in isolation.

**Supplementary Information:**

The online version contains supplementary material available at 10.1186/s12879-021-06376-1.

## Highlights

- Being able to use COVID-19 RT-PCR Ct values as simple markers of disease severity would allow for the easy and proactive identification and triaging of high-risk cases.
- In our cohort, we found no significant correlation between oxygen requirements on admission and RT-PCR Ct value.
- Higher viral loads of COVID-19 do not necessarily lead to a more severe disease presentation, indicating that other host-related factors may play a more important role.

## Introduction

Multidisciplinary and multi-angled efforts have been actively investigating the microbiologic and clinical characteristics of the novel coronavirus disease 2019 (COVID-19). One important angle is concerned with predictors of disease severity, which would help improve our understanding of the disease’s pathogenesis and the risk factors involved; both of which are of clinical and public health value. Despite its hypothetical plausibility, the role of viral load in the severity of respiratory infection is widely contested. In fact, within the same family of viruses, variations have been reported. In one study, the viral load of Influenza A/H3N2 was found not to be associated with hospitalization, in contrast to high viral loads of Influenza B [1]. In the case of COVID-19, contradictory information has similarly been reported [2]. The viral load of the patient can be deduced from the Cycle threshold (Ct) value of the reverse transcriptase polymerase chain reaction (RT-PCR) test conducted on the sample obtained. The RT-PCR test replicates the viral RNA from the patient’s sample until it is at a detectable concentration that exceeds the threshold value. The number of cycles necessary for that to take place is known as the Ct value. Thus, the lower the Ct value of a patient’s sample, the higher the viral load and vice versa.

## Methods

A retrospective cohort was conducted on randomly selected COVID-19 cases who were admitted to Ministry of Health Facilities between May and July 2020. Clinical data was collected from the selected cases. To examine the effect of Ct values on disease severity, collected clinical data was matched with laboratory data to find the patient’s Ct value on diagnosis (from May to July 2020). Clinical data of each case was connected to its respective Ct values upon admission. All cases were diagnosed as COVID-19 based on RT-PCR tests of nasopharyngeal samples. The majority of RT-PCR tests were conducted using Thermo Fisher Scientific (Waltham, MA) TaqPath 1-Step RT-qPCR Master Mix, CG (catalog number A15299) on the Applied Biosystems (Foster City, CA) 7500 Fast Dx RealTime PCR Instrument. The assay used followed the WHO protocol and targeted the E gene. If positive, the sample was confirmed by RdRP and N genes. The E gene CT value was reported and used in this study. CT Values > 40 were considered negative.

The reported clinical characteristics included: Comorbidities [sickle cell disease, glucose-6-phosphate dehydrogenase deficiency (G6PD), diabetes mellitus (DM), cardiovascular disease (CVD), hypertension, asthma, chronic obstructive pulmonary disease (COPD), obesity, chronic kidney disease (CKD)], medication regimen [kaletra, ribavirin, azithromycin, hydroxychloroquine, steroids, tocilizumab, and plasma infusion], and patient disease severity on admission.

Associations between Ct value and disease severity were statistically explored. A multivariable logistic regression model was used to control for confounders. The primary outcome investigated is disease severity as defined by requirement for supplemental oxygen on admission. The covariates adjusted for included sex, age, presentation, and comorbidities.

All methods were carried out in accordance with relevant guidelines and regulations, and informed consent obtained from all participants. In the case of dead patients, informed consent was obtained from next of kin, and of the guardian in the case of minors. The study was approved by the National COVID-19 Research and ethics committee, code number: CRT-COVID2020–082.

## Results

One thousand fifty-seven admitted cases were analysed, with a mean age of 46.26 (±16.68), and a male majority of (58.18%). Majority of the cohort (55.53%) was Bahraini. 66.70% of patients were symptomatic, with 44.75% presenting with cough, 22.99% with fever, 20.34% with dyspnea, and 10.97% with chest pain. With regards to comorbidities, most commonly reported included hypertension (29.61%), DM (27.91%), and G6PD deficiency (11.64%). This was followed by CVD (10.60%), asthma (4.73%), CKD (3.69%), and COPD (0.38%). A total of 18 (1.70%) patients had died.

**Fig. 1:**
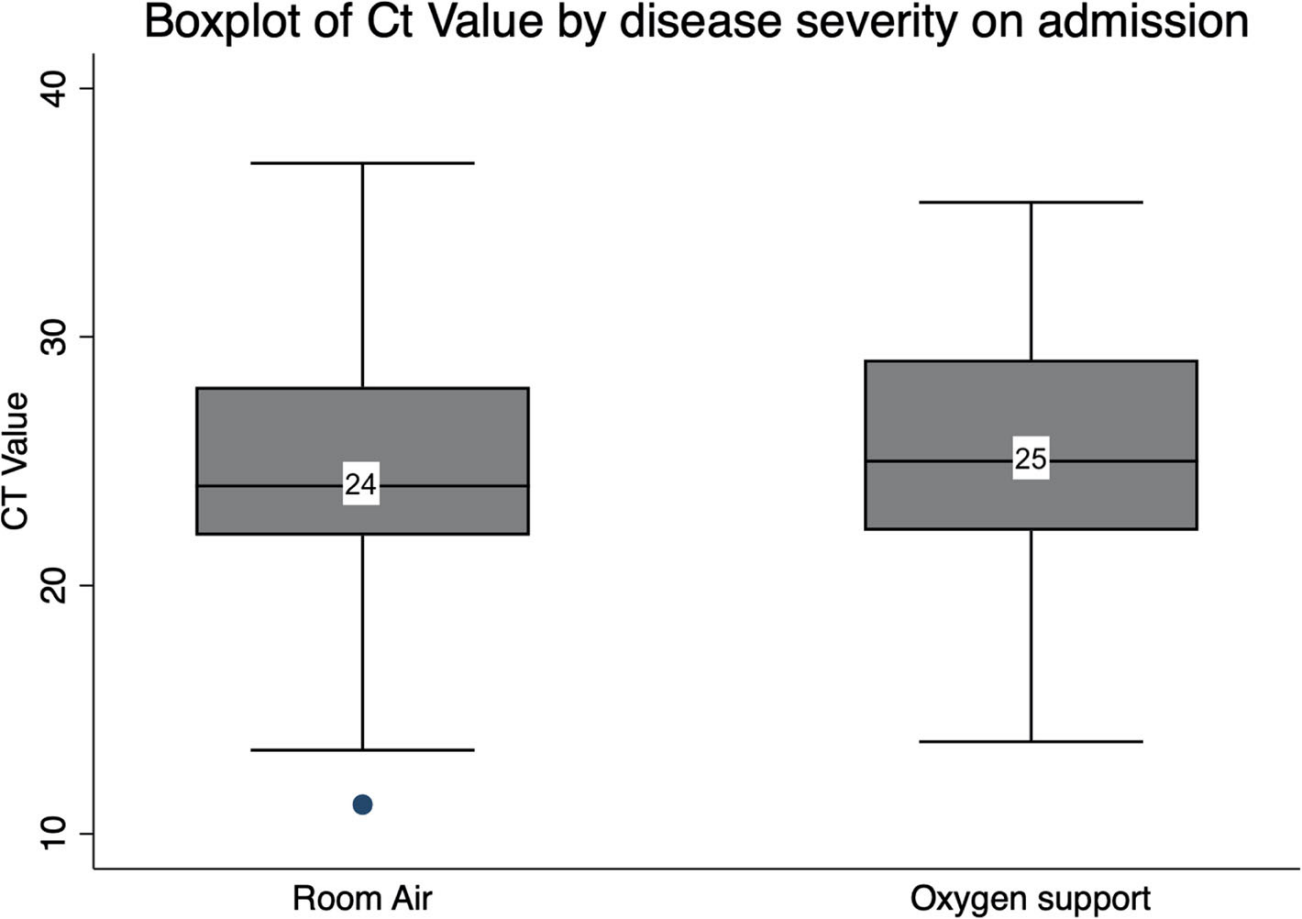
Boxplot of Ct Values by disease severity on admission

**Fig. 2:**
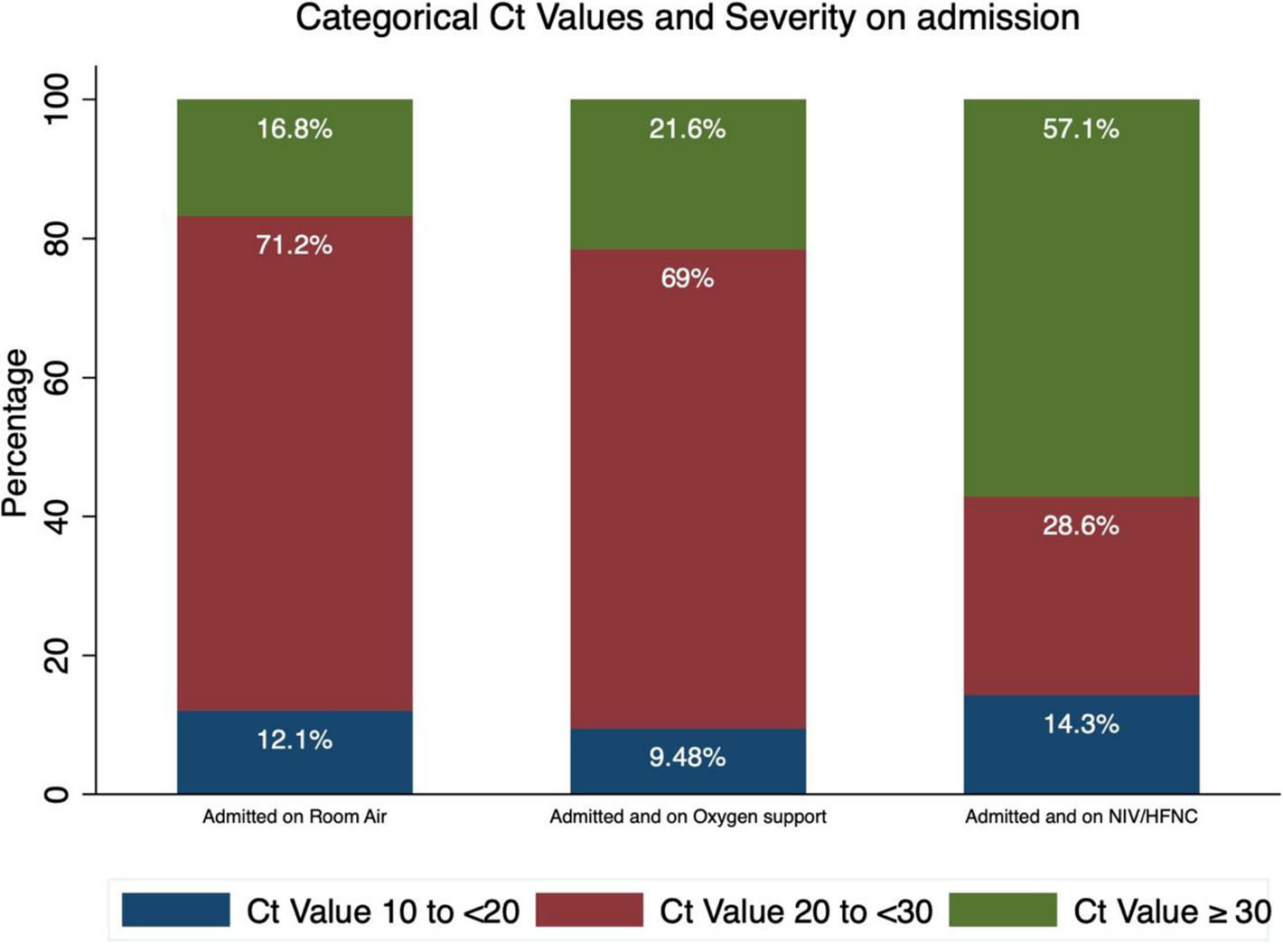
Percentage of Patients on Room Air, Supplemental Oxygen, and NIV/HFNC as per Ct Value Categories

The median Ct for patients admitted on room air was 24 (IQR 22–28), while patients admitted and required any form of oxygen support had a median Ct of 25 (IQR 22.1–29.1) (Fig. 1). Patients who were admitted on room air had a mean Ct value of 24.916, which was lower than patients admitted on supplemental oxygen (25.629), and non-invasive ventilation or high-flow nasal cannulation (NIV/HFNC) (27.039). Categorically, the highest percentage of patients with the lowest Ct value range of 10 to < 20 (highest viral load) was those on NIV/HFNC (14.3%). This was followed by patients on room air (12.1%), and those on supplemental oxygen (9.5%). As for the Ct value range of 20 to < 30, the majority of patients (71.2%) on room air fell within this category, as was the case for those on supplemental oxygen (69.0%). However, only 28.6% of severe cases on NIV/HFNC had a Ct value within that range. Instead, the majority of these cases (57.1%) had a Ct value of ≥30, while 21.6 and 16.8% of patients on supplemental oxygen and room air, fell within that range, respectively (Fig. 2).

After adjusting for confounders using a multivariate regression model, Ct value showed no statistical significance for an association with the requirement for oxygenation on admission (Odds ratio1.046, 95% CI 0.999 to 1.096, *p* = 0.054) (Supplemental 1).

## Discussion

In our cohort, we found no significant correlation between disease severity as indicated by respiratory indices, and viral load, as indicated by Ct values. As per a systematic review, eight (57%) out of 14 studies assessing a correlation between Ct value or viral loads and disease severity reported a significant association; our study would be the largest cohort (6 times the size of the largest one reported in the systematic review) to report no association [3]. Conversely, according to one study, patients with severe symptoms presented with 60 times higher viral load and prolonged viral shedding than patients with mild symptoms [4]. Additionally, a recent prospective cohort study showed an independent relationship between high viral load and mortality [5].

The fact that patients presenting more severely on admission relatively had both higher and lower Ct values in comparison to those presenting less severely may be more sensible when placed in the context of disease screening and time since symptom onset. Patients who were tested in our cohort were either contacts of positive cases, individuals with symptoms who got tested, or cases presenting to the Emergency Department. Patients presenting with severe cases on admission are likely to have progressed over a period of time while at home, before deciding that admission was necessary. This was specifically observed in the subpopulation of migrant workers, who formed a majority of our cohort in the initial months. In fact, our multivariate regression pointed out Bahraini’s to have significantly lower odds of presenting with severe disease on admission than non-Bahraini’s, which may support the hypothesised confounding nature of time since onset of symptoms rather than being an indicator of underlying biologic differences. As such, viral shedding may have been taking place for a longer period of time, thus leading to the patient’s Ct values being higher on admission compared to patients who only recently acquired the infection. The relationship between time since symptom onset and higher Ct values has already been well documented [6]. On the other hand, a study of 205 patients showed an inverse correlation between viral load and disease severity, which they also pointed out may have been a reflection of time from onset of infection [7]. Hence, time since symptom onset could be an important confounder when studying the association between Ct value and disease severity on admission. Nonetheless, without stratifying the two groups and accounting for bias and error in sample collection no such conclusion can be made with certainty. As such, although it may be the case that the correlation between viral load and disease severity is confounded by the time since symptom onset, it may also be that no inherent correlation exists. With a cohort of this size however, we can conclude that RT-PCR Ct values do not seem to be a viable metric to use as a simple, unprocessed, independent indicator of disease severity.

In the multivariate model, age was reported to be significantly associated with disease severity on presentation, which corroborates global findings [8]. Likewise, fever and shortness of breath also seem to indicate disease severity on admission, in terms of requirement for oxygen support. Overall, comorbidities (i.e. chronic diseases) have been established as an important prognostic indicator of disease severity, as we have shown in a previous study [9], and should be considered as a potential confounder when examining correlations with other variables. As such, they were controlled for in this study.

As mentioned earlier, our study suggests that RT-PCR Ct value as a simple unprocessed metric is not associated with disease severity on admission. More prospective studies of diverse cohorts are needed to shed light on this controversial topic.

## Supplementary Information

**Additional file 1: Supplemental Table 1.** Logistic regression (Outcome: Oxygen requirement on admission).

## Data Availability

The datasets used and/or analysed during the current study are available from the corresponding author on reasonable request.

## References

[1] Granados A, Peci A, McGeer A, Gubbay JB (2017). Influenza and rhinovirus viral load and disease severity in upper respiratory tract infections. J Clin Virol.

[2] Cho RH, To ZW, Yeung ZW, Tso EY, Fung KS, Chau SK, Leung EY, Hui TS, Tsang SW, Kung KN, Chow EY (2020). COVID-19 viral load in the severity of and recovery from olfactory and gustatory dysfunction. Laryngoscope.

[3] Rao SN, Manissero D, Steele VR, Pareja J (2020). A narrative systematic review of the clinical utility of cycle threshold values in the context of COVID-19. Infect Dis Ther.

[4] Liu Y, Yan LM, Wan L, Xiang TX, Le A, Liu JM, Peiris M, Poon LL, Zhang W (2020). Viral dynamics in mild and severe cases of COVID-19. Lancet Infect Dis.

[5] Pujadas E, Chaudhry F, McBride R, Richter F, Zhao S, Wajnberg A, Nadkarni G, Glicksberg BS, Houldsworth J, Cordon-Cardo C. SARS-CoV-2 viral load predicts COVID-19 mortality. Lancet Respir Med. 2020;8(9):e70. 10.1016/S2213-2600(20)30354-4 PMC7836878 32771081

[6] Singanayagam A, Patel M, Charlett A, Bernal JL, Saliba V, Ellis J, Ladhani S, Zambon M, Gopal R (2020). Duration of infectiousness and correlation with RT-PCR cycle threshold values in cases of COVID-19, England, January to may 2020. Eurosurveillance.

[7] Argyropoulos KV, Serrano A, Hu J, Black M, Feng X, Shen G, Call M, Kim MJ, Lytle A, Belovarac B, Vougiouklakis T (2020). Association of initial viral load in severe acute respiratory syndrome coronavirus 2 (SARS-CoV-2) patients with outcome and symptoms. Am J Pathol.

[8] Himmels JP, Borge TC, Brurberg KG, Gravningen KM, Feruglio SL, Berild JD (2021). COVID-19 and risk factors for hospital admission, severe disease and death–a rapid review, 3rd update.

[9] Mallah SI, Abdulrahman A, Alawadhi AI, AlQahtani MM. Clinical and epidemiological characteristics of COVID-19 in a multi-National Cohort in the Middle East. J Epidemiol Glob Health. 2021;11(2). 10.2991/jegh.k.210306.001. 10.2991/jegh.k.210306.001 PMC8242107 33876599

