# Supplementary material for: COVID-19 viral load not associated with disease severity: findings from a retrospective cohort study
Source: BMC Infect Dis. 2021 Jul 16;21:688. doi: 10.1186/s12879-021-06376-1 (PMC8284033; doi:10.1186/s12879-021-06376-1)
Supplement: Supplementary file 1 — Additional file 1: Supplemental Table 1. Logistic regression (Outcome: Oxygen requirement on admission). [file 12879_2021_6376_MOESM1_ESM.docx]

**COVID-19 Viral Load Not Associated with Disease Severity: Findings from a Retrospective Cohort Study**

AbdulKarim AbdulRahman^1,2^, Saad I. Mallah^3^, Manaf AlQahtani^1,3,4 *^

^1^National Taskforce for Combating the Coronavirus (COVID-19), Bahrain

^2^Mohammed Bin Khalifa Cardiac Centre, Bahrain
^3^Royal College of Surgeons in Ireland, Bahrain

^4^Bahrain Defence Force hospital, Bahrain

***Correspondence:**

***Lt. Col. Dr. Manaf Al-Qahtani***

MB Bch BAO(Ireland), MMM(USA), FACP(USA), FRCPC(Canada),

Head of Infection Control Unit and Microbiology Department, Bahrain Defence Force Hospital,

Chairperson of the COVID-19 Control Room,

Member of the National Taskforce for Combating the Coronavirus (COVID-19),

Associate Professor of Medicine, Royal College of Surgeons in Ireland – Bahrain.

**Email**: drmanaf@gmail.com

**Supplemental Table 1**

**Logistic regression**

Outcome: Oxygen requirement on admission

| Oxygenation requirement | Coef. | | St.Err. | t-value | | p-value | [95% Conf | | Interval] | Sig |
| --- | --- | --- | --- | --- | --- | --- | --- | --- | --- | --- |
| Ct value | 1.046 | | 0.025 | 1.93 | | 0.054 | 0.999 | | 1.096 | * |
| Age | 1.027 | | 0.008 | 3.39 | | 0.001 | 1.011 | | 1.043 | *** |
| Male | 0.768 | | 0.196 | -1.03 | | 0.301 | 0.466 | | 1.266 |  |
| Bahraini | 0.468 | | 0.121 | -2.93 | | 0.003 | 0.281 | | 0.778 | *** |
| COPD | 7.010 | | 10.341 | 1.32 | | 0.187 | 0.389 | | 126.295 |  |
| CKD | 3.481 | | 1.642 | 2.64 | | 0.008 | 1.381 | | 8.774 | *** |
| Symptoms | 2.460 | | 1.166 | 1.90 | | 0.058 | 0.971 | | 6.231 | * |
| Fever | 2.693 | | 0.635 | 4.20 | | 0.000 | 1.697 | | 4.275 | *** |
| Cough | 1.602 | | 0.416 | 1.81 | | 0.070 | 0.963 | | 2.664 | * |
| Sob | 5.350 | | 1.243 | 7.22 | | 0.000 | 3.393 | | 8.437 | *** |
| Constant | 0.002 | | 0.002 | -6.91 | | 0.000 | 0.000 | | 0.013 | *** |
|  | | | | | | | | | | |
| Mean dependent var | | 0.110 | | | SD dependent var | | | 0.313 | |  |
| Pseudo r-squared | | 0.234 | | | Number of obs | | | 1057.000 | |  |
| Chi-square | | 171.403 | | | Prob > chi2 | | | 0.000 | |  |
| Akaike crit. (AIC) | | 582.001 | | | Bayesian crit. (BIC) | | | 636.596 | |  |
| Area under ROC curve | | 0.84 | | | Number of observations | | | 1057 | |  |
|  | | | | | | | | | | |
| **** p<0.01, ** p<0.05, * p<0.1* | | | | | | | | | |  |
